# The complete chloroplast genome sequence of *Calanthe sieboldii* (orchidaceae)

**DOI:** 10.1080/23802359.2024.2324927

**Published:** 2024-03-04

**Authors:** Cuiying Peng, Dezhi Liao, Kun Liu, Xujun Wang, Wei Guo

**Affiliations:** a Hunan Academy of Forestry, Changsha, P. R. China; b Jindong Forest Farm of Yongzhou City, Yongzhou, P. R. China; c Taishan Academy of Forestry Sciences, Taian, P. R. China

**Keywords:** *Calanthe sieboldii*, chloroplast genome, Illumina sequencing, Orchidaceae

## Abstract

*Calanthe sieboldii* Decne. ex Regel is a terrestrial orchid with high ornamental and commercial value. In the present study, the chloroplast genome of *C. sieboldii* was characterized using Illumina technology. The chloroplast genome is 158,345 bp in length with a total AT content of 63.28%. There are 127 genes, comprising 37 tRNA genes, 82 protein-coding genes, and 8 rRNA genes. Phylogenetic relationship analysis was performed using common protein-coding genes extracted from 13 chloroplast genomes of Orchidaceae. It was revealed that *C. sieboldi* was sister to *C. hancockii* and closely clustered with *C. aristulifera* and *C. henryi*. These findings provide valuable genomic resources that are helpful for further phylogenetic and evolutionary studies of *Calanthe*.

## Introduction

*Calanthe sieboldii* Decne. ex Regel is a terrestrial orchid with bright yellow flowers emitting fragrance and has been cultivated as an excellent breeding parent with high ornamental and commercial value (Huang et al. 2022). It has a synonym of *Calanthe striata*. The native range of this species is China (Hunan), S. Korea, W. Central & S. Japan to SW. Taiwan (https://powo.science.kew.org/). It grows in the mountainous forests at altitudes from 1200 to 1500 m (Chen et al. 2009). The wild populations of *C. sieboldii* are tiny, which was categorized as a critically endangered species (National Forestry and Grassland Administration 2021). It has been classified as a national first-level key protected wild plant in China and has been registered in the Information System of Chinese Rare and Endangered Plants (ISCREP) (http://www.iplant.cn/rep). The chloroplast genome has been extensively studied in plant molecular evolution and systematics due to its small size and highly conserved genomic structure. Recent years, chloroplast genomes of *Calanthe* were sequenced for phylogenetic analysis (Chen et al. 2019; Miao et al. 2019; Zhong et al. 2019; Zhang et al. 2020). However, the chloroplast genome of *C. sieboldii* has not been reported yet. In this study, we used Illumina high-throughput sequencing technique to obtain the chloroplast genome of *C. sieboldii* and compared to other species in *Calanthe*. Our primary objectives were to provide a valuable genomic resource to study the features of the chloroplast genome of *C. sieboldii* and to determine its phylogenetic position.

## Materials and methods

**Figure 1.**
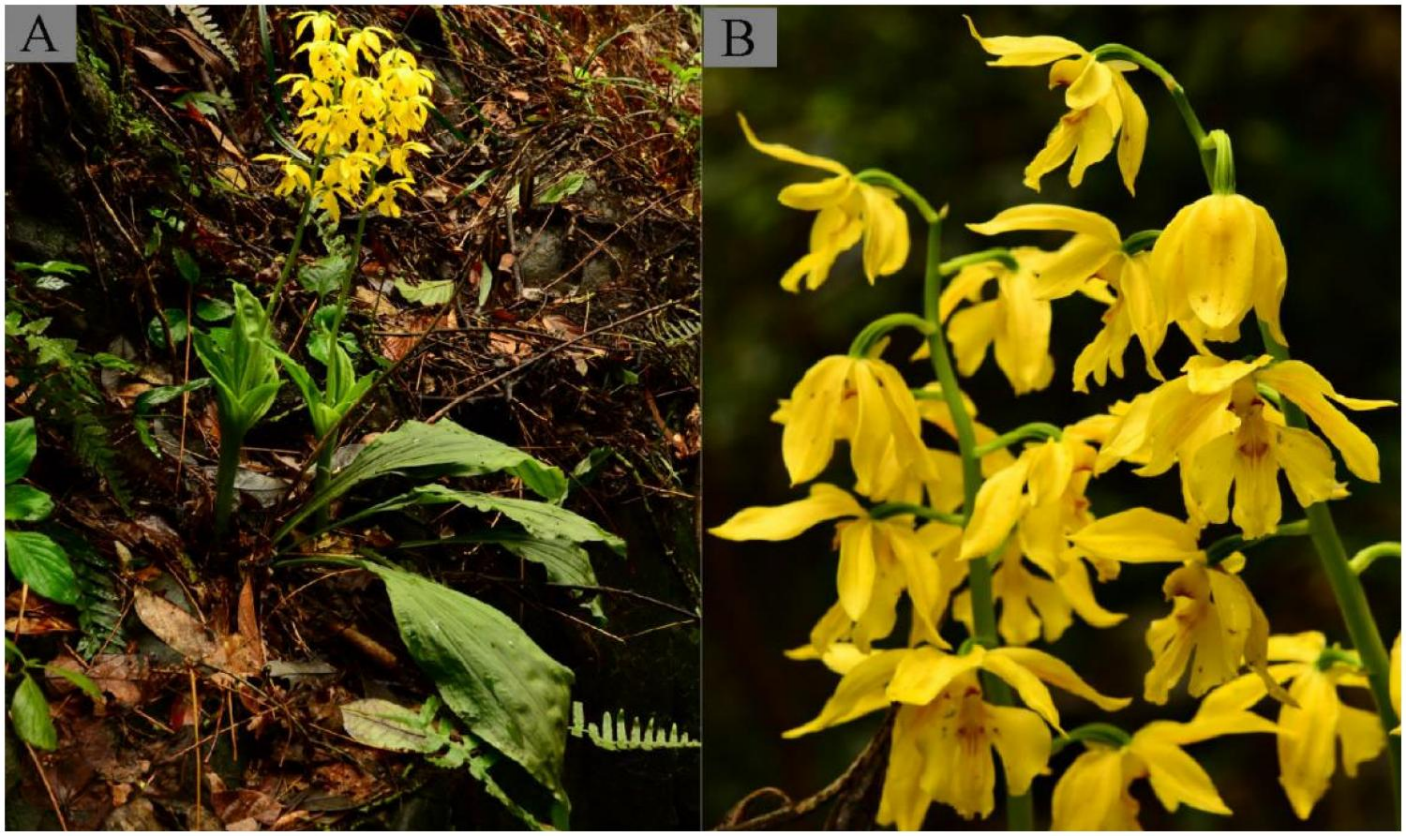
The morphological characteristics of *C. sieboldii.* (A) Habit, (B) Inflorescence. The photograph was taken by Xujun Wang from Jindong, Qiyang, Yongzhou, Hunan, P. R. China (26°17′56.62″ N, 112°05′35.84″ E). The most characteristic feature of the specimen: flowers bright yellow, large, slightly fleshy; dorsal sepal elliptic; petals narrowly elliptic; lip adnate to entire length of column wings, yellow, mottled red at base.

Plants of *C. sieboldii* were grown in Jindong, Qiyang, Yongzhou, Hunan, P. R. China (26°17′56.62″ N, 112°05′35.84″ E) (Figure 1). We rinsed the leaves quickly twice with sterile distilled water, then chilled them in a dry ice-ethanol bath. The voucher specimen (accession no. JDQ_2022_QYZ_CasHu) was deposited in a chest freezer at Hunan Academy of Forestry (HAF, Website: http://www.hnlky.cn/, Contact: Xujun Wang, E-mail: xjwang0514@sina.com).

We extracted total genomic DNA (gDNA) from 400 mg of leaf samples using the CTAB protocol (Doyle and Doyle 1987). The concentration, integrity and purity of the extracted gDNA was assessed using Qubit^®^ RNA Assay Kit in Qubit^®^ 3.0 Flurometer (Life Technologies, Carlsbad, CA, USA), agarose (1%) gel electrophoresis and NanoDrop 8000 spectrometer (Thermo Fisher Scientific, Waltham, MA, USA), respectively. Sonication was utilized to cleave the qualified gDNA into 350 bp fragments (Covaris, Woburn, MA, USA). The final library was obtained after DNA purification, terminal repair, A-tailing, adapter ligation and PCR amplification. Subsequently, a sequencing run was conducted on an Illumina HiSeq X platform (Illumina, San Diego, CA, USA). We got 90,472,192 clean reads and aligned the reads to the reference chloroplast genome of *C. hancockii* (GenBank: NC_064068). Finally, we assembled the chloroplast genome of *C. sieboldii* with the software SPAdes v3.11.1 (Bankevich et al. 2012) and annotated it with both of DOGMA (Wyman et al. 2004) and CPGAVAS (Liu et al. 2012) softwares. We extracted common protein-coding genes from the 13 complete chloroplast sequences through PhyloSuite (Zhang et al. 2020), then aligned them using MAFFT v7.313 (Katoh and Standley 2013) plugin integrated into PhyloSuite v1.2.1. A maximum likelihood phylogenetic tree was reconstructed using IQ-TREE v1.6.8 (Nguyen et al. 2015) under the GTR + F + I + G4 model for 5000 ultrafast bootstraps. Finally, the phylogenetic tree was visualized using FigTree v1.4.4.

## Results

**Figure 2.**
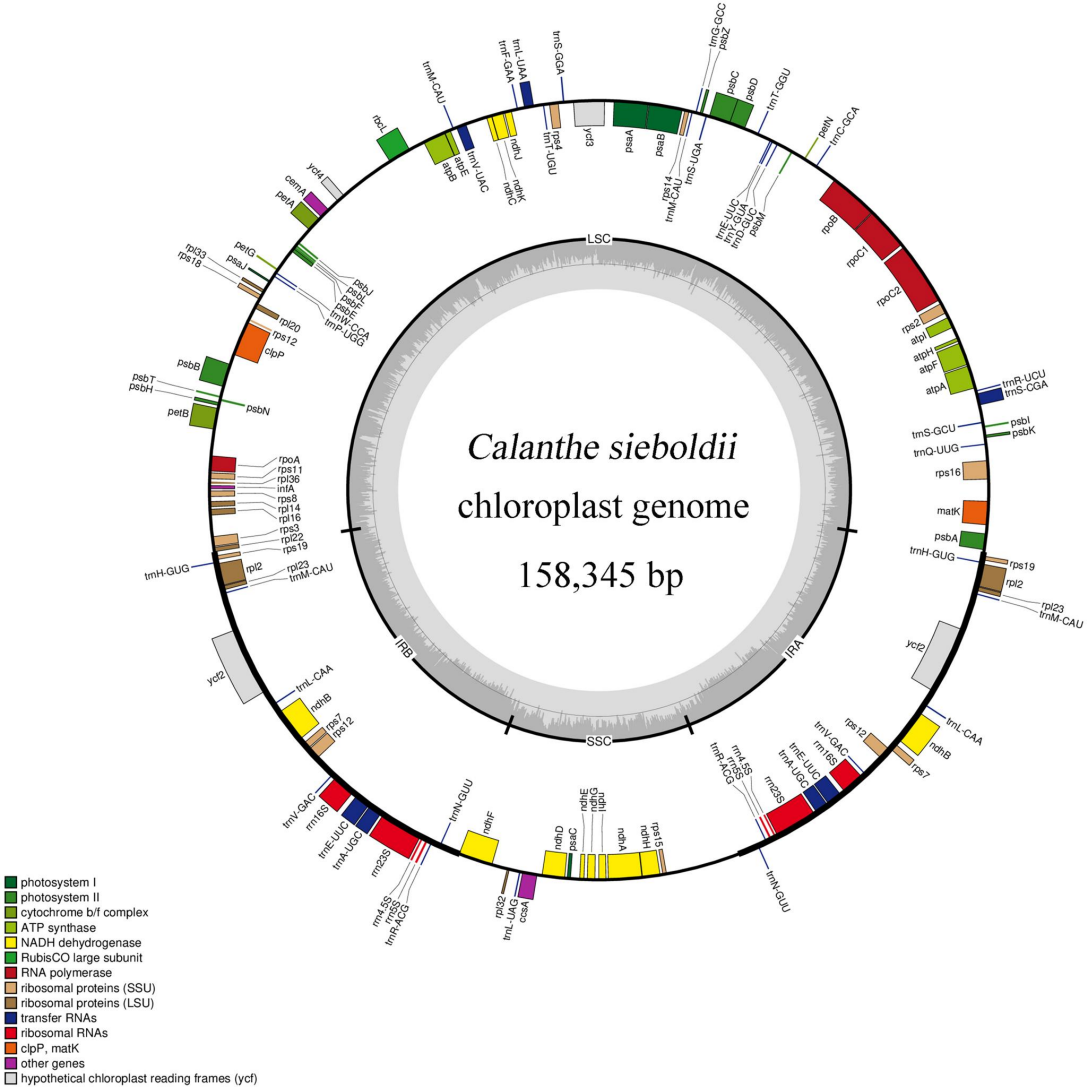
Circular map of the *C. sieboldii* chloroplast genome. Genes shown inside and outside the circle are transcribed counterclockwise and clockwise, respectively. Different colors represent genes with different types of function. The dark and light grey in the dashed area in the inner circle indicates the GC and at contents, respectively. LSC: large single-copy region; IR: inverted repeat; SSC: small single-copy region.

The average coverage depth of the assembly of the *C. sieboldii* chloroplast genome turned out to be 1,461 bp (Figure S1). The entire chloroplast genome (GenBank: OP270615) is 158,345 bp in size (Figure 2). It has a typical quadripartite structure, containing 87,204 bp large and 18,429 bp small single-copy regions with AT contents of 65.70 and 70.28%, respectively. The small and large single-copy regions are divided by two inverted repeat regions of 26,358 bp. Within the chloroplast genome, there are in total 127 genes, comprising 37 tRNA genes, 82 protein-coding genes, and 8 rRNA genes.

In total, we observed 18 intron-containing genes, comprising 16 genes (*rps16*, *trnS-CGA*, *atpF*, *rpoC1*, *trnL-UAA*, *trnV-UAC*, *petB*, *rpl2*, *ndhB*, *trnE-UUC*, *trnA-UGC*, *ndhA*, *trnA-UGC*_copy2, *trnE-UUC*_copy2, *ndhB*_copy2, *rpl2*_copy2), each containing one intron, and two genes (*ycf3* and *clpP*), each containing two introns (Figure S2). The single 5′-end of the trans-spliced gene *rps12* is positioned in the large single-copy region, while repeated 3′-end exons are located in the two inverted repeat regions (Figure S3).

**Figure 3.**
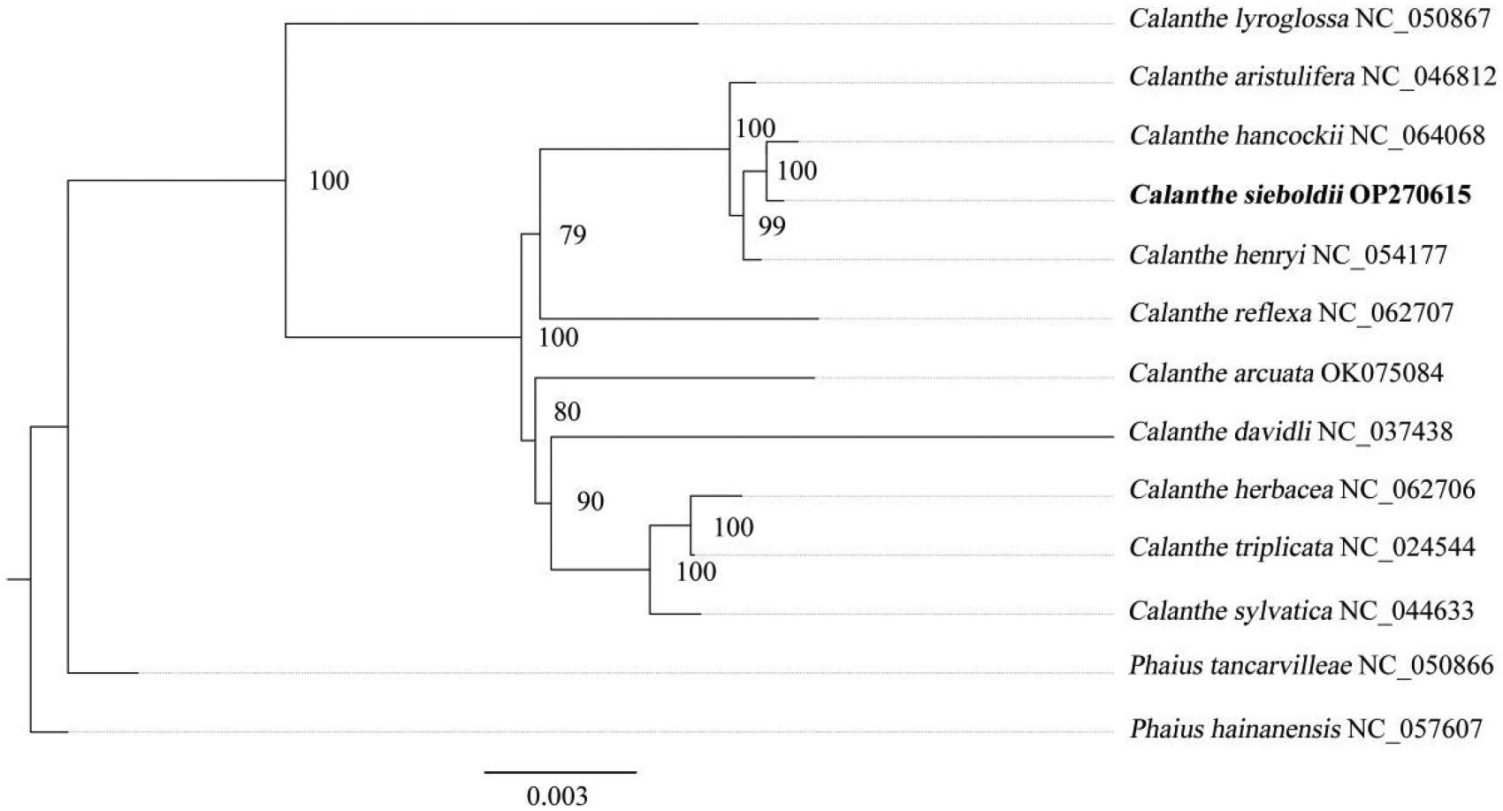
Phylogenetic tree based on common protein-coding genes of 13 species of the family orchidaceae. The following sequences were used: *C. sieboldii* OP270615 (this study), *C. hancockii* NC_064068 (unpublished), *C. aristulifera* NC_046812 (Ou et al. 2022), *C. henryi* NC_054177 (Ou et al. 2022), C. reflexa NC_062707 (unpublished), *C. davidli* NC_037438 (Miao et al. 2019), *C. sylvatica* NC_044633 (Ou et al. 2022), *C. herbacea* NC_062706 (unpublished), *C. triplicata* NC_024544 (Miao et al. 2019), *C. lyroglossa* NC_050867 (Ou et al. 2022), *phaius hainanensis* NC_057607 (Ou et al. 2022), *P. tancarvilleae* NC_050866 (Wang et al. 2021), C. arcuata OK075084 (unpublished). Supports for nodes were calculated via 1000 standard bootstrap replicates. Scale bar = 0.003.

To determine the phylogenetic position of *C. sieboldii*, a phylogenetic analysis was conducted using common protein-coding genes extracted from chloroplast genomes of 11 *Calanthe* and two *Phaius* species (Table S1). Two *Phaius* species were choosen as the outgroup because they were the closest genetic relatives of the *Calanthe*. The results revealed that the *C. sieboldi* is sister to *C. hancockii*, and they cluster together in a clade. The clade is closely associated with *C. henryi* and *C. aristulifera* (Figure 3).

## Discussion and conclusion

In this investigation, we successfully assembled the chloroplast genome sequence of *C. sieboldii* for the first time and provided annotations for it. It showed that the genome size, AT content and gene composition of the chloroplast genome sequence of *C. sieboldii* were similar to those of other species of the genus (Chen et al. 2019; Miao et al. 2019; Zhong et al. 2019; Zhang et al. 2020). Phylogenetic analysis demonstrated that *C. sieboldi* exhibited the closest relationship with *C. hancockii*. Overall, our study provides valuable genetic data for phylogenetic and evolutionary studies of the genus *Calanthe*.

## Supplementary Material

Supplemental Material

Supplemental Material

Supplemental Material

Supplemental Material

## Data Availability

The genome sequence data that support the findings of this study are openly available in GenBank of NCBI at (https://www.ncbi.nlm.nih.gov/) under accession no. OP270615. The associated BioProject, SRA, and Bio-Sample numbers are PRJNA872222, SRR21168988, and SAMN30449765, respectively.

## References

[CIT0001] Bankevich A, Nurk S, Antipov D, Gurevich AA, Dvorkin M, Kulikov AS, Lesin VM, Nikolenko SI, Pham S, Prjibelski AD, et al. 2012. SPAdes: a new genome assembly algorithm and its applications to single-cell sequencing. J Comput Biol. 19(5):455–477. doi:10.1089/cmb.2012.0021. 22506599 PMC3342519

[CIT0002] Chen XQ, et al. 2009. Typhaceae. In: Wu ZY, Raven PH, Hong DY, editors. Flora of China. Vol. 25. Orchidaceae. Beijing (China)/St. Louis (MI): Science Press/Missouri Botanical Garden Press; p. 292.

[CIT0003] Chen Y-Q, Lan S-R, Liu Z-J, Zhai J-W. 2019. The complete chloroplast genome sequence of *Calanthe delavayi* (Orchidaceae), an endemic to China. Mitochondr DNA B Resour. 4(1):1562–1563. doi:10.1080/23802359.2019.1601513.

[CIT0004] Doyle JJ, Doyle JD. 1987. A rapid DNA isolation procedure for small quantities of fresh leaf tissue. Phytochem Bull. 19(1):11–15.

[CIT0005] Huang M, Gao D, Lin L, Wang S, Xing S. 2022. Spatiotemporal dynamics and functional characteristics of the composition of the main fungal taxa in the root microhabitat of *Calanthe sieboldii* (Orchidaceae). BMC Plant Biol. 22(1):556. doi:10.1186/s12870-022-03940-y. 36456905 PMC9716840

[CIT0006] Katoh K, Standley DM. 2013. MAFFT multiple sequence alignment software version 7: improvements in performance and usability. Mol Biol Evol. 30(4):772–780. doi:10.1093/molbev/mst010. 23329690 PMC3603318

[CIT0007] Liu C, Shi L, Zhu Y, Chen H, Zhang J, Lin X, Guan X. 2012. CpGAVAS, an integrated web server for the annotation, visualization, analysis, and GenBank submission of completely sequenced chloroplast genome sequences. BMC Genomics. 13(1):715. doi:10.1186/1471-2164-13-715. 23256920 PMC3543216

[CIT0008] Miao L-Y, Hu C, Huang W-C, Jiang K. 2019. Chloroplast genome structure and phylogenetic position of *Calanthe sylvatica* (Thou.) Lindl. (Orchidaceae). Mitochondrial DNA B Resour. 4(2):2625–2626. doi:10.1080/23802359.2019.1642157. 33365654 PMC7706738

[CIT0009] National Forestry and Grassland Administration. 2021. The list of national key protected wild plants. https://www.gov.cn/zhengce/zhengceku/2021-09/09/content_5636409.htm.

[CIT0010] Nguyen LT, Schmidt HA, von Haeseler A, Minh BQ. 2015. IQ-TREE: a fast and effective stochastic algorithm for estimating maximum-likelihood phylogenies. Mol Biol Evol. 32(1):268–274. doi:10.1093/molbev/msu300. 25371430 PMC4271533

[CIT0011] Ou X-L, Qin Y, Zhang L-G, Sun T-G, Qin X-M. 2022. The complete chloroplast genome of the newly recorded species *Tainia acuminata* Averyanov (Orchidaceae) from China. Mitochondr DNA B Resour. 7(5):884–885. doi:10.1080/23802359.2022.2077666. PMC9176364 35692712

[CIT0012] Wang M, Yang J, Xue Q, Liu W, Niu Z, Ding X. 2021. The complete chloroplast genome sequence of *Oxystophyllum changjiangense* (Orchidaceae). Mitochondr DNA B Resour. 6(9):2638–2639. doi:10.1080/23802359.2021.1962766. PMC8366633 34409163

[CIT0013] Wyman SK, Jansen RK, Boore JL. 2004. Automatic annotation of organellar genomes with DOGMA. Bioinformatics. 20(17):3252–3255. doi:10.1093/bioinformatics/bth352. 15180927

[CIT0014] Zhang D, Gao F, Jakovlić I, Zou H, Zhang J, Li WX, Wang GT. 2020. PhyloSuite: an integrated and scalable desktop platform for streamlined molecular sequence data management and evolutionary phylogenetics studies. Mol Ecol Resour. 20(1):348–355. doi:10.1111/1755-0998.13096. 31599058

[CIT0015] Zhang S-D, Wang Q, Du M-M, Ling L-Z. 2020. Characterization of the chloroplast genome of *Calanthe henryi* (Epidendroideae; Orchidaceae). Mitochondrial DNA B Resour. 5(3):2273–2275. doi:10.1080/23802359.2020.1770141. 33367005 PMC7510761

[CIT0016] Zhong H, Shen L-M, Liu H-P, Liu Z-J, Wu S-S, Zhai J-W. 2019. The complete chloroplast genome of *Calanthe arcuata*, an endemic terrestrial orchid in China. Mitochondrial DNA B Resour. 4(2):2629–2630. doi:10.1080/23802359.2019.1639561. 33365656 PMC7687439

