# Supplementary material for: The complete chloroplast genome sequence of Calanthe sieboldii (orchidaceae)
Source: Mitochondrial DNA B Resour. 2024 Mar 4;9(3):314–7. doi: 10.1080/23802359.2024.2324927 (PMC10913714; doi:10.1080/23802359.2024.2324927)
Supplement: Supplemental Material [file TMDN_A_2324927_SM8930.docx]

Table S1. The protein-coding genes used for phylogenetic relationship analysis.

|  | **Gene name** |
| --- | --- |
| 1 | atpA |
| 2 | atpB |
| 3 | atpE |
| 4 | atpF |
| 5 | atpH |
| 6 | atpI |
| 7 | ccsA |
| 8 | cemA |
| 9 | clpP |
| 10 | infA |
| 11 | matK |
| 12 | ndhA |
| 13 | ndhB |
| 14 | ndhB_copy2 |
| 15 | ndhC |
| 16 | ndhD |
| 17 | ndhE |
| 18 | ndhF |
| 19 | ndhH |
| 20 | ndhI |
| 21 | ndhJ |
| 22 | ndhK |
| 23 | petA |
| 24 | petB |
| 25 | petD |
| 26 | petG |
| 27 | petN |
| 28 | psaA |
| 29 | psaB |
| 30 | psaC |
| 31 | psaJ |
| 32 | psbA |
| 33 | psbB |
| 34 | psbC |
| 35 | psbD |
| 36 | psbE |
| 37 | psbF |
| 38 | psbH |
| 39 | psbI |
| 40 | psbJ |
| 41 | psbK |
| 42 | psbL |
| 43 | psbM |
| 44 | psbN |
| 45 | psbT |
| 46 | psbZ |
| 47 | rbcL |
| 48 | rpl14 |
| 49 | rpl16 |
| 50 | rpl2 |
| 51 | rpl20 |
| 52 | rpl22 |
| 53 | rpl23 |
| 54 | rpl23_copy2 |
| 55 | rpl2_copy2 |
| 56 | rpl32 |
| 57 | rpl33 |
| 58 | rpl36 |
| 59 | rpoA |
| 60 | rpoB |
| 61 | rpoC1 |
| 62 | rpoC2 |
| 63 | rps11 |
| 64 | rps12 |
| 65 | rps14 |
| 66 | rps15 |
| 67 | rps16 |
| 68 | rps18 |
| 69 | rps19 |
| 70 | rps19_copy2 |
| 71 | rps2 |
| 72 | rps3 |
| 73 | rps4 |
| 74 | rps7 |
| 75 | rps7_copy2 |
| 76 | rps8 |
| 77 | ycf2 |
| 78 | ycf2_copy2 |
| 79 | ycf3 |
| 80 | ycf4 |
