# Supplementary material for: The complete chloroplast genome sequence of Calanthe sieboldii (orchidaceae)
Source: Mitochondrial DNA B Resour. 2024 Mar 4;9(3):314–7. doi: 10.1080/23802359.2024.2324927 (PMC10913714; doi:10.1080/23802359.2024.2324927)
Supplement: Supplemental Material [file TMDN_A_2324927_SM8929.pdf]

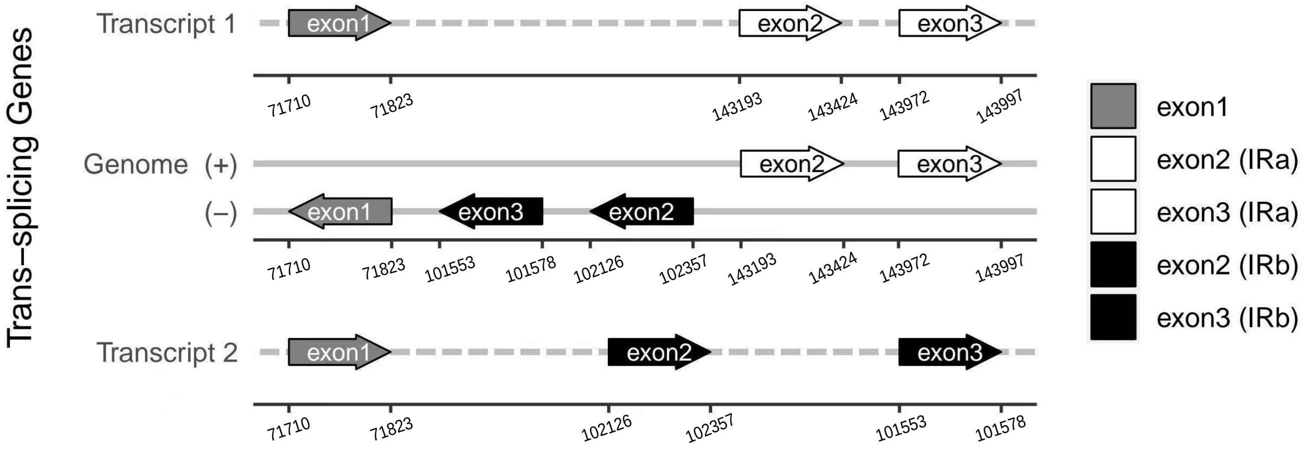

Figure S3. Schematic maps of the trans-splicing gene *rps12* in the chloroplast genome of *C. sieboldii*. The direction of the genes is represented by arrows. The maps were constructed using CPGview.
