# Supplementary material for: The complete chloroplast genome sequence of Calanthe sieboldii (orchidaceae)
Source: Mitochondrial DNA B Resour. 2024 Mar 4;9(3):314–7. doi: 10.1080/23802359.2024.2324927 (PMC10913714; doi:10.1080/23802359.2024.2324927)
Supplement: Supplemental Material [file TMDN_A_2324927_SM8928.pdf]

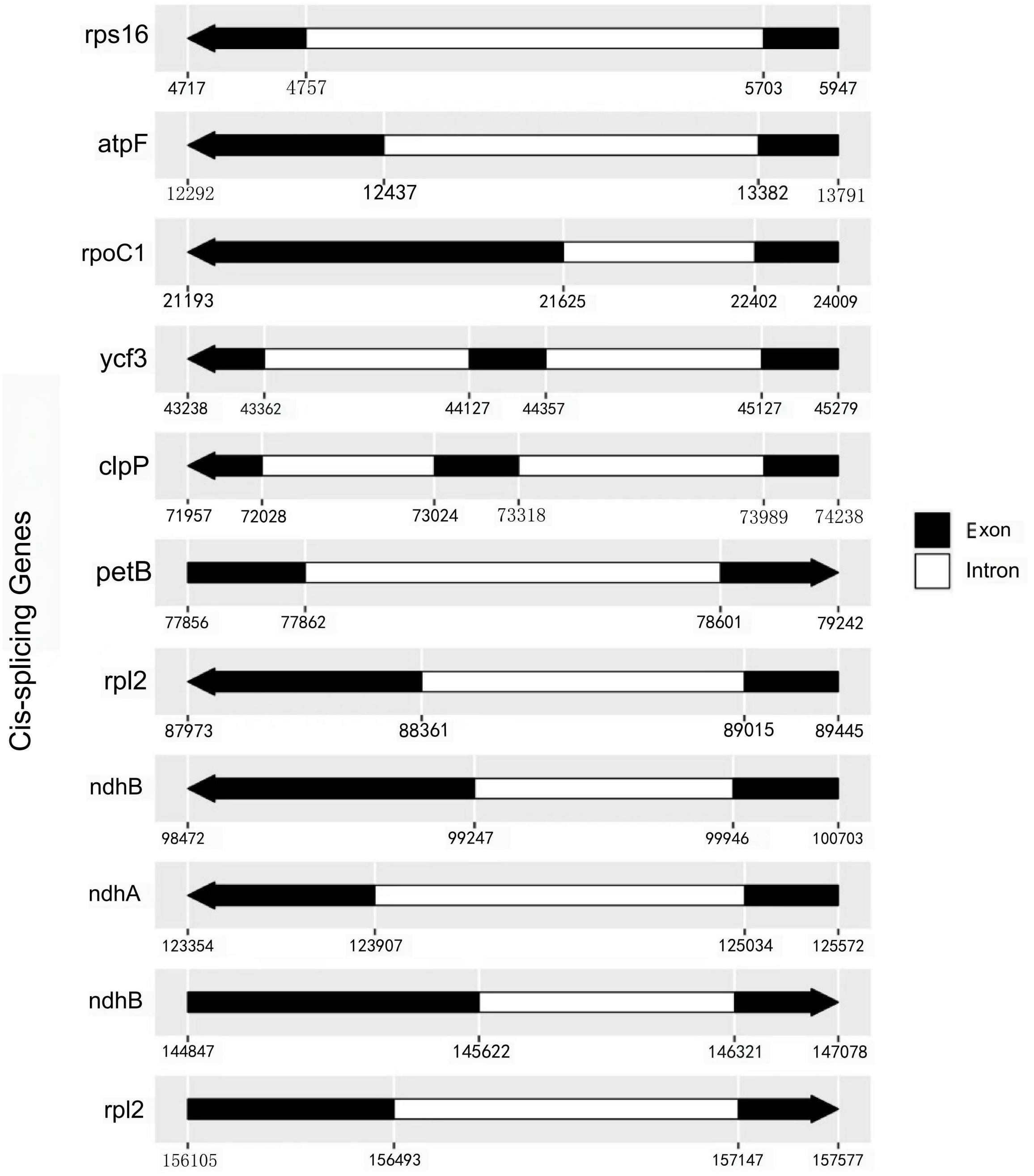

Figure S2. Schematic maps of the cis-splicing genes in the chloroplast genome of *C. sieboldii*. Exons and introns are shown in black and white, respectively. The gene names are shown on the left. The direction of the genes is represented by arrows. The maps were constructed using CPGview.
