# Supplementary material for: The complete chloroplast genome sequence of Calanthe sieboldii (orchidaceae)
Source: Mitochondrial DNA B Resour. 2024 Mar 4;9(3):314–7. doi: 10.1080/23802359.2024.2324927 (PMC10913714; doi:10.1080/23802359.2024.2324927)
Supplement: Supplemental Material [file TMDN_A_2324927_SM8927.pdf]

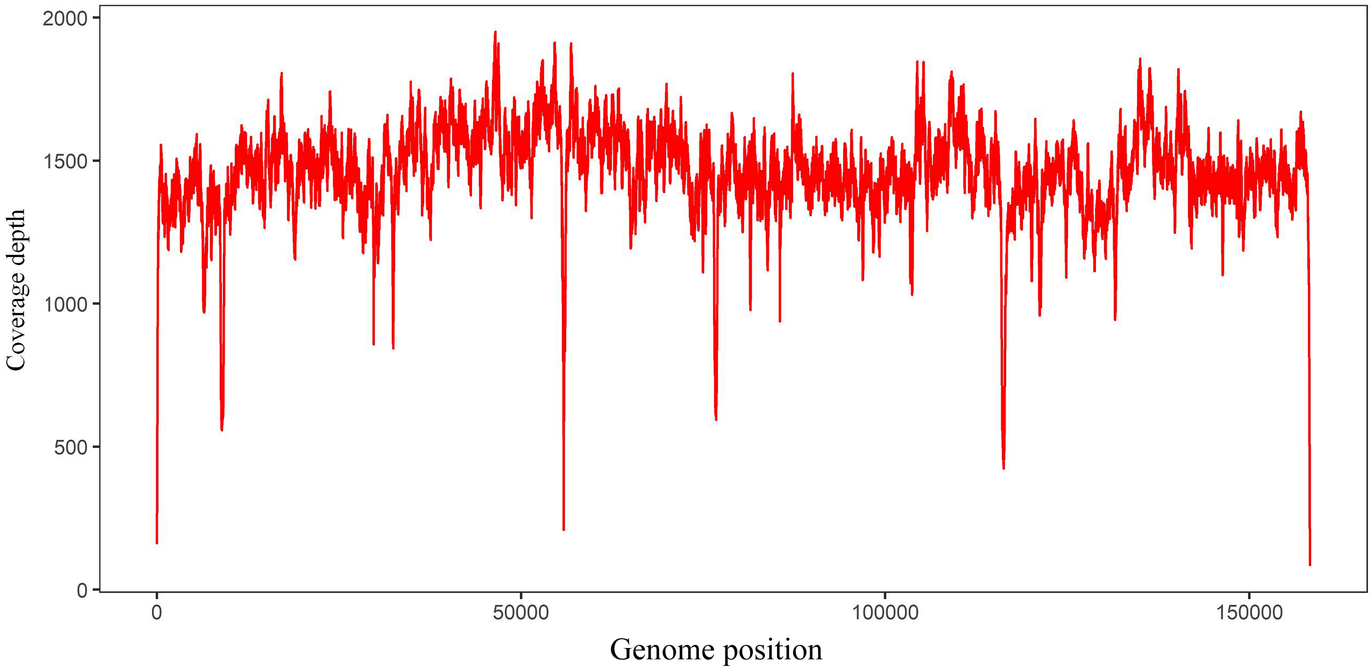

Figure S1. The coverage depth of the chloroplast genome of *C. sieboldii*. The map was generated using BWA by aligning sequencing data onto the genome. The coverage depth for each nucleotide ranged from 83 to 1951 with an average of 1461.
